# Randomised controlled trial of an automated, interactive telephone intervention (TLC Diabetes) to improve type 2 diabetes management: baseline findings and six-month outcomes

**DOI:** 10.1186/1471-2458-12-602

**Published:** 2012-08-03

**Authors:** Emily D Williams, Dominique Bird, Andrew W Forbes, Anthony Russell, Susan Ash, Robert Friedman, Paul A Scuffham, Brian Oldenburg

**Affiliations:** 1 School of Public Health and Preventive Medicine, Monash University, Melbourne, Australia; 2 School of Medicine, University of Queensland, Brisbane, Australia; 3 Department of Diabetes and Endocrinology, Princess Alexandra Hospital, Brisbane, Australia; 4 Institute of Health and Biomedical Innovation, Queensland University of Technology, Queensland, Australia; 5 School of Exercise and Nutrition Sciences, Queensland University of Technology, Brisbane, Australia; 6 Centre for Applied Health Economics, School of Medicine, Griffith Health Institute, Griffith University, Queensland, Australia

## Abstract

**Background:**

Effective self-management of diabetes is essential for the reduction of diabetes-related complications, as global rates of diabetes escalate.

**Methods:**

Randomised controlled trial. Adults with type 2 diabetes (n = 120), with HbA_1c_ greater than or equal to 7.5 %, were randomly allocated (4 × 4 block randomised block design) to receive an automated, interactive telephone-delivered management intervention or usual routine care. Baseline sociodemographic, behavioural and medical history data were collected by self-administered questionnaires and biological data were obtained during hospital appointments. Health-related quality of life (HRQL) was measured using the SF-36.

**Results:**

The mean age of participants was 57.4 (SD 8.3), 63% of whom were male. There were no differences in demographic, socioeconomic and behavioural variables between the study arms at baseline. Over the six-month period from baseline, participants receiving the Australian TLC (Telephone-Linked Care) Diabetes program showed a 0.8% decrease in geometric mean HbA_1c_ from 8.7% to 7.9%, compared with a 0.2% HbA_1c_ reduction (8.9% to 8.7%) in the usual care arm (p = 0.002). There was also a significant improvement in mental HRQL, with a mean increase of 1.9 in the intervention arm, while the usual care arm decreased by 0.8 (p = 0.007). No significant improvements in physical HRQL were observed.

**Conclusions:**

These analyses indicate the efficacy of the Australian TLC Diabetes program with clinically significant post-intervention improvements in both glycaemic control and mental HRQL. These observed improvements, if supported and maintained by an ongoing program such as this, could significantly reduce diabetes-related complications in the longer term. Given the accessibility and feasibility of this kind of program, it has strong potential for providing effective, ongoing support to many individuals with diabetes in the future.

## Background

The rapid increase in rates of diabetes poses a significant public health problem globally. Diabetes is currently estimated to affect 285 million adults worldwide, with the prevalence predicted to rise to 438 million by the year 2030 [1]. Its complications contribute significantly to ill health, disability, poor quality of life and premature death. The associated global economic burden is projected to reach at least US$376 billion in 2030 [2]. Although guidelines and targets for optimal diabetes management are well documented [3], it is estimated that 40% of individuals with diabetes have sub-optimal glycaemic control [4,5], significantly increasing their risk of costly and debilitating diabetes-related complications [6,7].

Diabetes self-management education facilitates the acquisition of knowledge and skills to improve disease management and has been found to improve glycaemic control [8], with program duration being a critical predictor of this success [9]. Providing ongoing and long-term diabetes management support, particularly to those people living in rural and remote areas, is a major challenge for all health systems around the world. This highlights the need to develop and evaluate more feasible, accessible ways of providing such support for large numbers of people with diabetes than is traditionally offered. Using information and communication technology (ICT) to provide diabetes management education and support directly to patients offers such potential, by overcoming many of the barriers associated with more traditional modes of program delivery. Use of ICT has been shown to yield improvements in self-care knowledge and behaviour of patients and clinical outcomes associated with the prevention and control of chronic health conditions, including diabetes [10-12]. Some studies have evaluated the role of automated or semi-automated telephone-delivered diabetes management interventions on glycaemic control, however, the results have been inconsistent with varying levels of reliance upon health professionals [13-15].

The Telephone-Linked Care (TLC) program is an automated and interactive telephone system designed to emulate telephone encounters between patients and health professionals [16] and to complement standard medical care. TLC systems have been previously used to effectively screen people with specific health conditions [17,18], promote self-care behaviours [19-22] and provide monitoring of and feedback to patients with a range of chronic diseases [23-26].

A randomised controlled trial was conducted to evaluate a TLC program - the Australian TLC Diabetes program - designed to improve type 2 diabetes management. This paper presents the six-month results for the study’s primary outcomes, glycosylated haemoglobin and health-related quality of life (HRQL), and it also describes the sample baseline characteristics, compared with a large Australian population study.

## Methods

### Study design

The study methodology has been detailed elsewhere [27]. In brief, the study was a two-arm prospective randomised controlled trial, with adults with type 2 diabetes randomised to either the intervention (Australian TLC Diabetes program) arm or ‘usual care’ control arm. Data were collected between July 2008 and December 2010. Ethics approval was received from the Human Research Ethics Committees for all collaborating hospitals and Monash University.

### Participant recruitment and randomisation

Participants were recruited through advertisements in newspapers, flyers distributed to health professionals and to members of Diabetes Australia – Queensland, community newsletters and through diabetes clinics at three major hospitals in Brisbane (Princess Alexandra Hospital, Royal Brisbane and Women’s Hospital, and Prince Charles Hospital).

There were two steps to the eligibility screening (Table 1). In the first step, which took place during the initial contact via telephone or in person, research staff excluded individuals who did not meet all of the Step 1 eligibility criteria or who met any of the Step 1 exclusion criteria. If potentially eligible, participants attended a baseline appointment at either Princess Alexandra or Royal Brisbane and Women’s Hospital, where full information was provided, informed consent was obtained and baseline data collected. At that appointment, baseline questionnaires were completed and fasting blood specimens were taken, along with other clinical data (blood pressure, weight, height and waist circumference). Blood tests were conducted by Queensland Pathology using standardised assays. The second screening step verified the glycosylated haemoglobin (HbA_1c_) inclusion criterion (≥ 7.5%). The final sample included 120 adults; n = 60 in each of the study arms. The allocation ratio was 1:1 and the allocation sequence was computer-generated. The arm allocation was conducted using a 4x4 block randomised block design with the participant as the unit of randomisation.

**Table 1:** Inclusion and exclusion criteria for study recruitment

**Inclusion criteria**	**Exclusion criteria**
*Eligibility Step 1*	
Type 2 diabetes diagnosis of ≥ 3 months	Diagnosed with dementia/psychiatric co-morbidity
Aged 18–70 years	Currently enrolled in another intervention trial
Residing in greater Brisbane area, Australia	Undergone bariatric surgery in past 2 years
Stable diabetes pharmacotherapy type for ≥ 3 months	Pregnant, lactating, or planning to become pregnant within next 12 months
Ability to clearly speak/understand English via telephone	Diagnosed with condition likely to be fatal within 1 year
Stable pharmacotherapy dosage for ≥ 4 weeks	
Weekly access to telephone	
*Eligibility Step 2*	
HbA_1c_ ≥ 7.5%	

### Study arms

All participants received a quarterly newsletter containing general health information; this aimed to maintain participation in both arms. Participants in both arms were advised to continue with their usual medical care. The usual care arm received no further intervention. The treating physicians were not blinded to the allocation.

### Intervention arm

#### Australian TLC Diabetes program

The intervention took place over six months during which they received the Australian TLC Diabetes program. Its main component is the Telephone-Linked Care (TLC) Diabetes system, an automated interactive telephone system, developed collaboratively by the Australian research team and researchers at the Medical Information Systems Unit, Boston University, USA. The Australian TLC Diabetes system has been designed to improve diabetes management by targeting the following key self-management behaviours: blood glucose testing, nutrition, physical activity and medication-taking. Users were asked to call the system weekly using a landline or mobile phone. TLC’s responses, including feedback and encouragement, were tailored according to information entered in the TLC database at the start and the answers that it received from participants during all calls.

#### Training to use the TLC system

The TLC Coordinator met with participants within one week of their baseline data collection to instruct them on the use of the TLC Diabetes kit (containing the TLC Handbook, an ACCU-CHEK® Advantage glucose meter, test strips, and a Bluetooth™ device for uploading their blood glucose results to the TLC Diabetes system). For current smokers, a smoking cessation information pack was also provided. During this session, participants completed a training call to the TLC Diabetes system. Participants were asked to conduct all blood glucose self-monitoring with the study glucose meter and to upload its readings immediately preceding their weekly telephone conversations with the TLC system. Each participant chose a unique personal password that they keyed in at the start of each call that linked the call to their database file and ensured correct participant identification and confidentiality. Before the participants' first call to the TLC system, the TLC Coordinator obtained self-care clinical targets for the participants from their primary healthcare provider (including recommended number of weekly blood glucose tests and blood glucose range, and clearance for physical activity).

#### Content of weekly telephone calls

Participants were requested to make weekly calls to the system over six months, with calls lasting five-20 minutes, depending upon the call content and participant responses. Blood glucose monitoring was the first topic covered in each weekly call. It was followed by one of three other topics, with these being medication-taking, physical activity or healthy eating (calls 9 to 12 and 21 to 24). When diabetes medication was not prescribed, the medication-taking topic was replaced with physical activity. When clearance for physical activity was not provided by the patient’s treating physician, physical activity was replaced by medication-taking. In cases when there was no clearance for physical activity and no pharmaceutical treatment of diabetes, the participant did not hear a second topic on some calls.

#### TLC Coordinator

The TLC Coordinator briefly telephoned participants after the first two calls and at weeks six, 12, and 20, to identify and resolve any technical issues with the TLC Diabetes system or to determine reasons for not calling. In addition, the TLC Diabetes system sent email "alerts" to a dedicated study email address if any unusual clinical or other issues arose during the conversations, for example, where there were two or more hypoglycaemic levels in the past week. In this instance, the Coordinator would advise the participant of the importance of visiting their primary care physician. More detail on the intervention is available elsewhere [27].

### Measurement

Participants in both arms completed comprehensive clinical and self-report assessments at baseline (Time 1), six months following baseline (Time 2), and at 12 months (Time 3); this paper presents the baseline characteristics and six-month primary outcome findings.

### Outcome variables

The primary outcomes were HbA_1c_ measured by fasting blood tests taken at the hospital appointment, and HRQL assessed by the participants’ self-completion of the SF-36 version 2 (divided into mental and physical component summary scores) [28].

**Figure 1:**
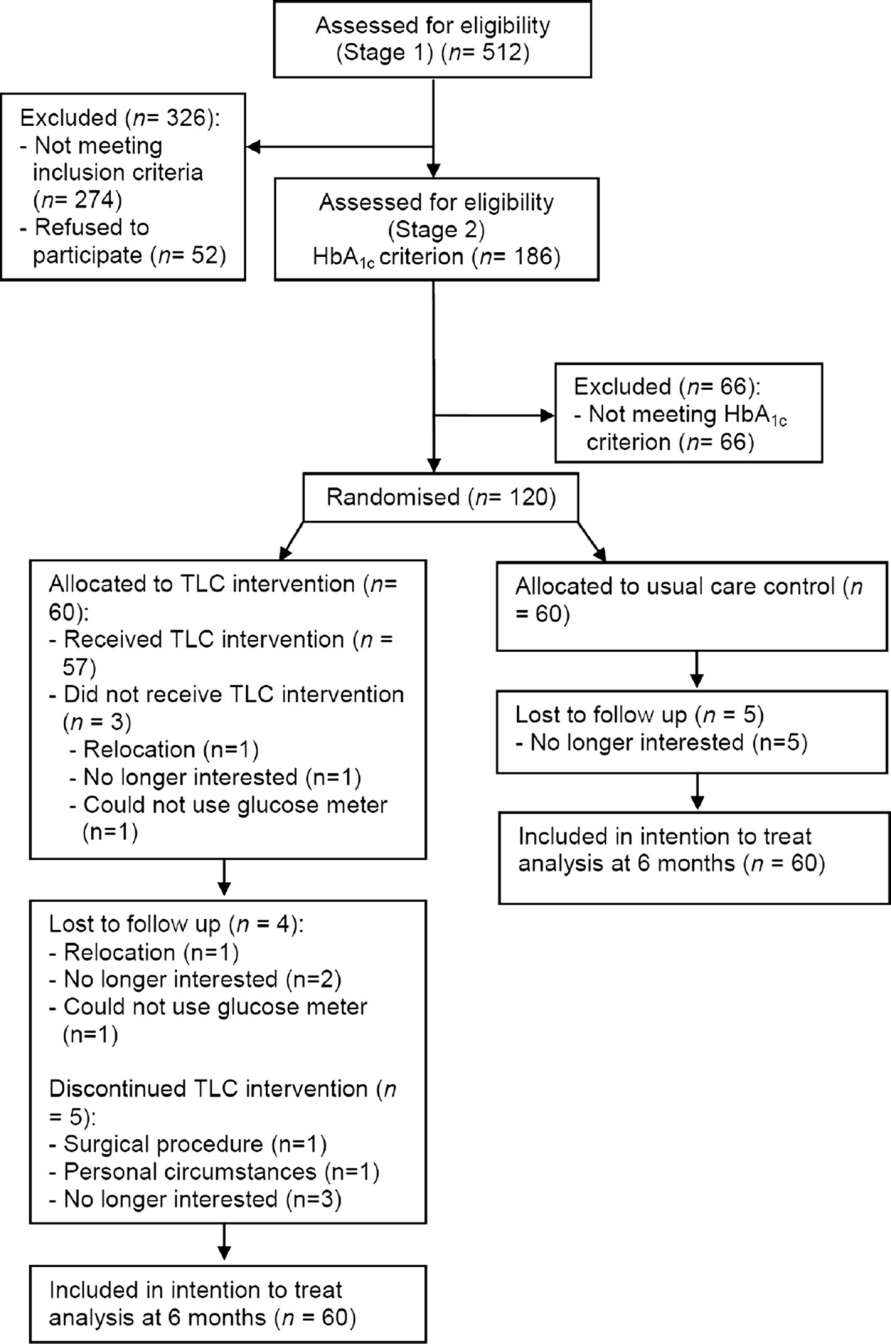
Participant flow diagram.

Figure 1 illustrates the stages of recruitment and randomisation.

### Representativeness of study sample (Table 2)

**Table 2:** Baseline characteristics of Australian Telephone-Linked Care (TLC) Diabetes sample

		**TLC Diabetes Intervention (n = 60)**	**Usual care (n = 60)**	**Total TLC sample (n = 120)**	**AusDiab sub-sample (n = 156)**
*Demographic variables*					
Age		58.4 (8.2)	56.4 (8.3)	57.4 (8.3)	56.6 (8.8)
Sex	% male	61.7	63.3	62.5	59.8
Country of birth	% born in Australia	71.7	68.3	70.0	66.7
Marital status	% cohabiting	75.4	74.6	75.0	67.9
Employment	% working	46.7	45.0	45.8	47.7
income	% > $40,000	46.7	51.7	49.2	
Education	% > secondary school	60	70	65.0	55.8
Private medical insurance	% with	56.7	55.0	55.8	47.3
Psychosocial risk factors					
Depression	Low	66.7	78.3	72.5	-
	Intermediate	26.7	20.0	23.3	
	High	6.7	1.7	4.2	
Anxiety	Low	90.0	88.3	89.2	-
	Intermediate	6.7	8.3	7.5	
	High	3.3	3.3	3.3	
Social support	% low	20.0	21.7	20.8	NC
Nutritional self-efficacy		15.2 (3.0)	15.0 (3.2)	15.1 (3.1)	NC
Physical activity self-efficacy		12.8 (3.3)	12.7 (3.5)	12.7 (3.4)	NC
HRQL	Physical component summary	43.7 (8.4)	43.8 (10.2)	43.6 (9.3)	45.2 (12.7)
	Mental component summary	49.8 (8.7)	49.5 (9.1)	49.6 (8.9)	49.5 (9.8)
Health behaviours					
Smoking status	% never	51.7	53.3	52.5	34.0
	% ex-smoker	45.0	46.7	45.0	45.5
	% current	3.3	0	1.7	17.9*
Physical activity	% none	5.0	5.2	5.1	22.4
	% do not meet guidelines	35.0	43.1	39.0	31.4
	% meet guidelines	60.0	51.7	55.9	46.2*
Diet (n = 110)	Energy (kJ/day) ^‡^	7658 (5884–9745)	7811 (6080–9566)	7704 (6025–9638)	7467 (5850–9455)
	Fibre (g/day) ^‡^	23 (18–29)	23 (17–29)	23 (17–29)	23 (17–30)
	Fat (g/day) ^‡^	73 (53–93)	76 (63–95)	75 (57–94)	71 (56–94)
	Saturated fat (g/day) ^‡^	27 (21–37)	30 (23–38)	29 (22–38)	28 (22–38)
Self-care					
	% adherence to blood glucose testing				
	recommendations	40.0	30.0	36.2	NC
	% checked feet everyday	31.7	20.0	26.1	NC
	% insulin/diabetes medical adherence everyday	87.9	86.0	84.0	NC
Self-reported health	% ≥ good	74.9	73.3	74.2	65.8
*Medication use*					
Inject insulin	% on insulin	41.7	45.0	43.3	NC
*Clinical measures*					
Systolic blood pressure (mmHg)		135.4 (15.0)	137.0 (15.0)	136.2 (15.0)	140.2 (18.9)*
Diastolic blood pressure (mmHg)		32.5 (28.7-35.9)	32.9 (29.2-37.8)	33.6 (28.8-36.9)	30.0 (26.5-34.6)*
Body mass index (kg/m^2^) ^‡^		107.7 (100.0-114.6)	113.1 (101.7-122.4)	111.0 (101.5-118.7)	103.2 (92.0-115.2)*
Waist circumference (cm) ^‡^		8.6 (8.0-9.2)	8.5 (7.9-9.5)	8.5 (7.9-9.3)	8.8 (8.1-9.8)*
Glycosylated haemoglobin (HbA_1c_) (%)^‡^		9.8 (8.4-11.1)	9.5 (8.1-12.0)	9.6 (8.2-11.4)	11.5 (9.8-14.1)*
Fasting glucose (mmol/l) ^‡^		15.0 (9.6-24.0)	13.0 (8.3-22.8)	14.0 (9.1-23.8)	NC
Fasting insulin (mU/l) ^‡^		2.3 (1.4-3.6)	1.9 (1.2-3.0)	2.2 (1.3-3.3)	NC
HOMA Insulin Resistance ^‡^		4.0 (3.5-4.9)	4.0 (3.4-5.2)	4.0 (3.5-5.2)	5.6 (4.8-6.3) *
Total cholesterol (mmol/l) ^‡^		32.5 (28.7-35.9)	32.9 (29.2-37.8)	33.6 (28.8-36.9)	30.0 (26.5-34.6)*
High density lipoprotein cholesterol (mmol/l) ^‡^		1.0 (0.8-1.1)	1.0 (0.8-1.2)	1.0 (0.8-1.1)	1.2 (1.0-1.4) *
Low density lipoprotein cholesterol (mmol/l) ^‡^		2.1 (1.8-3.1)	2.2 (1.6-3.0)	2.1 (1.7-3.0)	3.2 (2.7-3.9)*
Triglycerides (mmol/l) ^‡^		1.6 (1–2.1)	1.5 (1.1-2.0)	1.5 (1.1-2.1)	2.0 (1.3-2.9) *
Creatinine (μmol) ^‡^		83.0 (64.8-98.5)	73.0 (62.0-86.8)	78.0 (62.8-95.3)	83.5 (73.0-92.8)*
Estimated glomerular filtration rate (ml/min) ^‡ †^		76.0 (64.0-91.0)	85.5 (77.3-91.0)*	83.0 (70.8-91.0)	78.1 (69.5-87.7)*
*Clinical history (self-report)*					
Doctor-diagnosed hypertension (%)		63.3	68.3	65.8	46.8
Doctor-diagnosed hypercholesterolaemia (%)		60.0	66.7	63.3	48.0
Doctor-diagnosed diabetic eye complications (%)		15.0	21.7	18.3	NC
Doctor-diagnosed diabetic neuropathy (%)		18.3	25.0	21.7	NC
Doctor-diagnosed kidney disease (%)		11.7	5.0	8.3	NC
Doctor-diagnosed cardiovascular disease (%)		28.3	30.0	29.2	NC

Data are presented as means (SD) and percentages, or as ^‡^medians (inter-quartile range) for skewed data. Group comparisons between TLC study arms and between TLC and AusDiab samples of normally distributed data used independent samples t-tests and chi square tests. Group comparisons between TLC study arms and between TLC and AusDiab samples of non-normally distributed variables used Mann–Whitney U test, *p <0.05.

^†^ Estimated glomerular filtration rate data highly skewed (values over 90 ml/min labelled 91).

HRQL: Health-related quality of life.

HOMA: Homeostasis model assessment.

High risk AusDiab group inclusion criteria are type 2 diabetes, within TLC age-range, and HbA_1c_ ≥ 7.5 %; TLC-AusDiab group comparison are made with full TLC sample (n = 120).

NC: Not comparable - missing comparisons with AusDiab subsample due to incomparable methods of data collection between studies.

To examine the representativeness of the Australian TLC Diabetes sample, the baseline characteristics were compared with data from the Australian Diabetes, Obesity and Lifestyle (AusDiab) study [29], the largest national, population-based sample of Australians measuring the overall prevalence of diabetes and other chronic conditions. The AusDiab baseline study was conducted during 1999–2000 with data from 11,247 adults [29]. Demographic and behavioural data were collected during interview, and diabetes status was assessed using fasting plasma glucose and oral glucose tolerance tests. A subsample of this nationally representative study, those identified as having diabetes (and based on TLC inclusion criteria), provides the best comparison for the TLC study sample.

### Statistical analyses

Detailed power calculations were described in an earlier paper [27], indicating the need to recruit 340 participants to detect a small clinical change of 0.4% in HbA_1c_ with 90% power assuming a 30% rate of loss to follow-up. However, due to the slowness of recruitment (described below), our final sample comprised a total of 120 participants (60 per study arm). Although the comprehensive recruitment effort achieved a very good response from individuals with diabetes (n = 512), a large proportion of these respondents either did not wish to participate or were ineligible due to either not meeting the HbA_1c_ or age eligibility criteria. Recruitment was stopped after 18 months with 120 participants having been recruited. Therefore, the power calculations were re-evaluated based on this number of participants, again assuming 30% loss to follow-up. With 80% power and a type 1 error of 5% (two-tailed), a difference in our primary outcome, HbA_1c_, of 0.61% between the intervention and control arms (based on a standard deviation change of 1.0% between the randomised arms) at 12-month follow-up can be detected. This effect size would indicate a feasible outcome of clinical significance [30] for the intervention.

For the analysis of the six-month results, HbA_1c_ values were logarithmically transformed in order to achieve an approximate normal distribution. Analyses of covariance were used to examine the effects of the intervention (study arm allocation) on the primary outcomes (log HbA_1c_ and HRQL), with the inclusion of baseline values of the outcomes as covariates. Results for HbA_1c_ are presented as geometric means for each study arm and as a ratio of geometric means when comparing study arms. The geometric mean is a natural quantity to use for presenting the centre of skewed data and is computed by exponentiating the average of the logarithmically transformed HbA_1c_ values [31]. To assess heterogeneity of the effect of TLC according to baseline values, interactions between study arm allocation and baseline values were included in further regression models. Creatinine and e-GFR were included as covariates in these analyses, since their levels at baseline differed sizeably between study arms. The sensitivity of conclusions to imbalances in baseline characteristics was assessed via additional ANCOVA analyses adjusting for all characteristics exhibiting any potentially important imbalances. To account for subjects lost to follow-up in intention-to-treat analyses, multiple imputation was performed using ten imputed datasets [32].

For the comparison of the baseline TLC sample characteristics with the AusDiab study sample, as well as for the attrition comparisons, independent samples t-tests (continuous data) and chi-square tests (categorical data) were used where the data were normally distributed, and Mann–Whitney U tests were employed for highly skewed data. All analyses were performed using SPSS 18.0, with the statistical significance level set at p < 0.05.

## Results

Of the 52 individuals who did not wish to participate at the initial eligibility screening stage, the primary reason for non-participation was lack of interest (n = 21), with an additional 11 reporting potential difficulties with travel for the baseline data collection. Other reasons included lack of time due to work and other commitments. There were no age differences between those who were willing and unwilling to participate, although there was a higher proportion of women who were unwilling to participate compared with those who chose to participate (61.5% compared with 43%).

As shown in Table 2, which summarises the baseline characteristics of the TLC and usual care arms, the Australian TLC Diabetes sample had a mean age of 57.4 years (± 8.3), with a higher proportion of men (62.5%) than women. The vast majority of participants were born in Australia (70.0%), were married or cohabiting with a partner (75.0%), with education above secondary school level (65.0%). Approximately half of the sample were employed (45.8%) and had complementary private medical insurance (55.8%). The mean number of hours per week spent exercising was reported to be 6.1 (± 6.4), with the majority of the sample (55.9%) participating in the nationally-recommended level of weekly physical activity (>150 minutes of exercise per week in at least 5 sessions per week [33]). Only 1.7% of the sample were current smokers. Approximately three quarters of the sample rated their health as good or higher (74.2%). Nearly two-thirds of the sample had been previously diagnosed by a doctor with hypertension (65.8%) and hypercholesterolaemia (63.3%), and therefore were likely to be receiving treatment for these conditions as was reflected in their blood pressure and lipid profiles that predominantly fell within the normal range.

### Comparison of baseline sample characteristics between study arms

The baseline sample characteristics were compared across the usual care and intervention arms to evaluate the randomisation process (Table 2). Comparison of the baseline characteristics across usual care and intervention arms revealed important differences in e-GFR, which showed a significantly greater impairment in renal function in the intervention compared with usual care arm, and creatinine. Other differences observed were in age, education, and self-care behaviours (adherence to blood glucose testing recommendations and daily insulin/diabetes medications, and foot inspections). Adjustments were made for these variables in sensitivity analyses.

### Post-intervention results at six months

#### Attrition

Of the total sample, 92.5% completed the six-month assessment (see Figure 1). Overall, nine participants (two women and seven men) withdrew from participation in the study, four in the intervention arm and five in the usual care arm. The reasons given for withdrawal from the usual care arm were all related to frustration at ‘missing out’ on the intervention. The participants receiving the Australian TLC Diabetes intervention withdrew for a range of reasons, including relocation, being unable to use the blood glucose meter, and disappointment with the intervention. The sociodemographic, behavioural or biological profiles were compared between those people who remained in the study and the nine people who withdrew. There were no significant differences at baseline across any of the domains of risk factor profiles.

#### Use of Australian TLC Diabetes system

The mean number of completed calls for the Australian TLC Diabetes participants during the six-month intervention was 18 (± 6), ranging between 2 and 27 calls, with a mean call duration of 11 minutes (± 1). The mean percentage of completed calls out of the expected weekly calls for all individuals in the intervention condition was 76% (± 22). More detailed analyses of the usage of the Australian TLC Diabetes system are beyond the scope of this paper and are to be presented in a future manuscript.

A small number of people in the intervention arm (n = 5) discontinued participation in the intervention but still completed the six-month assessment (Figure 1). Out of these, two participants made less than five calls and one made only seven calls.

#### Study outcomes

These analyses were based on intention-to-treat. There was a statistically significant difference in HbA_1c_ at six months between the usual care and TLC Diabetes arms. The geometric mean (arithmetic means provided in parentheses) of HbA_1c_ decreased from 8.7% (8.8%) to 7.9% (8.0%) in the TLC Diabetes arm, compared with 8.9% (9.0%) to 8.7% (8.9%) in the usual care arm, with the adjusted ratio of six-month geometric means of 0.91 (95% CI 0.86-0.93, p = 0.002) (Table 3). The ratio of 0.91 means that the geometric mean HbA_1c_ at six months in the TLC arm is 0.91 of the value in the usual care arm after adjustment for baseline covariates. There was slight evidence that the difference in HbA_1c_ at six months between study arms increased with baseline HbA_1c_ (*p* = 0.09 for the interaction term in regression model). This suggested that the difference in six-month HbA_1c_ between TLC and usual care patients was greater in patients with high baseline HbA_1c_ values than in patients with low values. Of participants in the intervention arm, 20 % achieved HbA_1c_ levels of 7.0% or lower (95% CI 9.6-29.7), compared with 15% (95% CI 4.4-24.7) in the usual care arm (*p* = 0.32).

**Table 3:** Baseline and post-intervention primary outcome values between usual care and Australian TLC Diabetes arms

	**Baseline n = 60**	**Post-intervention n = 60**	Difference between groups* (95% CI, *p)*
**HbA**_**1c**_**(%)**			Ratio
Usual care	8.9 (8.6-9.2)	8.7 (8.7-9.0)	0.91 (0.86-0.93, *p* = 0.002)
TLC Diabetes	8.7 (8.4-9.0)	7.9 (7.6-8.3)	
**Health-related quality of life - mental**			
Usual care	49.5 (47.1-50.3)	48.7 (47.1-50.3)	3.0 (0.8-5.2 *p* = 0.007)
TLC Diabetes	49.8 (47.5-52.0)	51.7 (50.2-53.3)	
**Health-related quality of life - physical**			
Usual care	45.4 (43.0-47.9)	45.2 (43.8-46.6)	0.4 (−1.7-2.4, *p* = 0.7)
TLC Diabetes	45.5 (43.0-47.9)	45.6 (44.1-47.0)	

Data presented in the first two columns are geometric means (95 % CI) for HbA_1c_ values and arithmetic means (95 % CI) for HRQL values. The post-intervention values are adjusted for baseline values, e-GFR and creatinine.

*The result in the last column for HbA_1c_ is the ratio of the geometric means in the TLC Diabetes arm compared with usual care arm.

For HRQL, it is the difference in arithmetic means. All analyses were conducted based on the intention-to-treat principle and adjust for the baseline of the outcome variable, e-GFR and creatinine values.

In terms of HRQL, the mental component summary score was found to be significantly different between the two arms at six months (difference = 3.0, *p* = 0.007), after controlling for baseline mental HRQL, plus other covariates (Table 3). Mental HRQL improved in the TLC Diabetes group, compared with those in the usual care group where mental HRQL decreased marginally. There was no interaction between study arm allocation and baseline levels for mental HRQL (*p* = 0.4). No differences were observed in physical HRQL between the usual care and intervention arms (*p* = 0.7).

#### *Comparison of sample characteristics between* Australian *TLC and AusDiab samples*

To determine the representativeness of the TLC sample at baseline, we used a comparable subsample of individuals from the AusDiab study, obtained from applying the Australian TLC Diabetes criteria for age range and HbA_1c_ levels (≥ 7.5%) to the subsample (n = 643) of those classified in AusDiab as having diabetes. 156 AusDiab participants were identified for comparison with the Australian TLC Diabetes sample. Overall, the AusDiab and TLC samples were similar (Table 2). There were no significant differences between the TLC sample and the AusDiab subsample across demographic variables, HRQL, and self-reported health variables. Behaviourally, there were no differences in nutrition self-reports between the study populations, however the TLC sample reported markedly lower smoking rates and were more likely to perform the recommended levels of exercise. In terms of their clinical profiles, the TLC sample appeared healthier, with lower systolic blood pressure, and generally better glucose and lipid profiles. These results, however, are likely to reflect the increased levels of doctor-diagnosed hypertension and hypercholesterolaemia, and therefore probably high levels of treatment in the TLC sample. Interestingly, despite their reported healthier behavioural profiles, the TLC sample were significantly more likely to be obese using both BMI and waist circumference classifications.

## Discussion

This randomised controlled trial evaluated the efficacy of an automated, interactive telephone intervention for improving the management of diabetes. As far as we are aware, this is one of the first studies in the world to formally evaluate an automated telephone system for diabetes management that involves tailoring to individual needs and the findings offer promising results for the longer term use of this kind of program for people with diabetes. We have demonstrated that the Australian TLC Diabetes program significantly improved glycaemic control and mental HRQL after six months for those who participated in the program compared with the routine care condition.

Participation in the Australian TLC Diabetes intervention led to a significant improvement of HbA_1c_, compared with the routine care available to people with diabetes in Brisbane, Australia. The mean reduction in HbA_1c_ of 0.8 % in the intervention arm is of substantial clinical significance if maintained long-term. Results from the UKPDS study highlight the substantial reductions in all diabetes endpoints associated with 1% reduction in HbA_1c_[7], such as 21% of deaths related to diabetes, 14% of myocardial infarction and 37% microvascular complications [30]. A meta-analysis reported comparable levels of HbA_1c_ improvement from the pooled effects of 31 previous interventions providing education on self-management of diabetes [9]. The majority of studies cited in the review, however, directly involved healthcare professionals/health workers for the provision of diabetes management education. Another meta-analysis evaluating the use of mobile phone interventions to improve glycaemic control showed a pooled change of 0.5% over six months, however, again with heavy involvement of healthcare personnel for intervention delivery [11]. One previous study of another fully-automated telephone intervention aimed at improving glycaemic control failed to show significant post-intervention differences between intervention and control groups in levels of HbA_1c_[13]; however, that system did not provide tailored feedback to individuals. Therefore, a major advantage of the Australian TLC Diabetes program is its successful impact on glycaemic control and the potential for reduced costs and increased accessibility associated with an automated telephone-linked system for the provision of tailored diabetes management.

In addition to the observed improvements in glycaemic control, mental HRQL was significantly enhanced in people who received the intervention compared with those who did not, despite this not being a specific focus of the TLC program for the trial. The burden of daily management of diabetes and the development of complications lead to compromised HRQL in populations with diabetes [34,35], and therefore enhancing well-being, in addition to diabetes management per se, is an additionally important outcome. Despite this improvement reflecting only a small effect size (0.20) [36], the literature in this field indicates that even small effect sizes of HRQL improvement may be of clinical significance in the longer term [37-39]. Interestingly, the physical component of HRQL did not improve during the six-month intervention period. A brief computer-assisted diabetes self-management intervention on quality of life outcomes showed no change in HRQL, however, their two-month follow-up might not have been long enough to detect changes [40]. In contrast, the pooled results from 20 publications showed that people with diabetes experience improved HRQL after receiving interventions designed to develop their diabetes self-management behaviours [37], although this meta-analysis did not differentiate between the mental and physical components of HRQL.

Another important aspect of this study is the focus on people with poor glycaemic control (HbA_1c_ ≥ 7.5%), indicating difficulty in their self-management of diabetes with the available routine care. These people are likely to be most at risk of the development of complications associated with diabetes, and therefore, given the results achieved, Australian TLC Diabetes has the potential to improve the health of the highest risk groups. Consequently, this program also provides the opportunity to significantly reduce the financial burden of type 2 diabetes on the healthcare system. Subsequent analyses will examine the cost-effectiveness of the program, which will have important implications for the widespread implementation of the program.

Our comparison of the TLC sample with a ‘matched’ subgroup from the AusDiab study sample suggests that the TLC participants did not differ significantly in terms of demographic characteristics from the best available data from a general population-based diabetes sample in Australia. The baseline AusDiab study, conducted in 1999–2000, offers benchmark national data on the prevalence of diabetes, obesity, hypertension, and kidney disease in Australia. This indicates the representativeness and external validity of our results and their applicability to other diabetes populations.

The trial was completed in accordance with the Medical Research Council’s guidelines for the effective design and evaluation of complex intervention trials [41]. Principal components of any effective complex intervention include feasibility, participant-engagement, identification of mechanisms for intervention outcomes, and trial fidelity [42]. The feasibility and relevance of the Australian TLC Diabetes program are demonstrable within the current context of type 2 diabetes. The accessibility of the telephone-delivered intervention over the long-term is particularly important for a widespread chronic condition, such as diabetes, which requires ongoing management and affects a large proportion of the population. The very high usage of the Australian TLC Diabetes system and results to date indicate that the participants in the intervention arm engaged with the program, with over three quarters of weekly calls being completed. Full details of system usage were recorded as part of the data collection and will be reported elsewhere for full process evaluation of the system’s usability and participant satisfaction, as well as whether the cost of the intervention provides acceptable value for money. Furthermore, the intervention was able to affect pathways that led to improvements in glycosylated haemoglobin and therefore diabetes management, as well as improvement in mental health-related quality of life for the participants. The fidelity of the trial implementation in accordance with the original design and protocol [27] was strong. Difficulties were encountered during recruitment and this led to increased recruitment opportunities via enhanced presence at Diabetes Australia – Queensland shops and seminars and hospital diabetes clinics. The sample size was smaller than originally planned, however, as discussed, the sample obtained is powered to detect group differences that will be both statistically and clinically significant at 12-month follow-up. No changes were applied regarding the randomisation process or implementation of the intervention.

Although only glomerular filtration rate significantly varied across the study arms at baseline, other baseline characteristics (Table 2) showed some differences. Separate analyses tested the impact of the inclusion of these variables individually on the main results and the main outcome results did not change. As with most research, it is possible that a selection bias operated in this study, with people willing to participate being more likely to prioritise their health and/or have the social, educational, and economic resources to accommodate participation. The study requirement of access to a telephone meant that there may have been a socioeconomic selection bias; however in the geographic area from which we recruited, over 96% of households have a fixed phone connection, so we are confident that this criterion did not appreciably influence participation. It is also possible that the reduced sample size and some of the challenges associated with trial recruitment may limit generalisability. More research is required to investigate generalisability and to explore uptake by others with diabetes. Although there was a suggestion of an increasing effect of intervention with increasing baseline HbA_1c_ values (from the interaction test), this did not reach conventional levels of statistical significance and should be reassessed in future studies.

A substantial body of research conducted over the last 30 years has drawn attention to the importance of ongoing support and follow-up to sustain improvements in diabetes management and management of other chronic conditions, with strong links to health and self-care behaviours [43-45]. Therefore a diabetes management support program such as this, designed to provide easy access to long-term (potentially cost-effective) support, is of paramount importance, and hence, this kind of program also requires detailed evaluation in the longer term as well. A subsequent paper will elucidate the changes in behaviour that may have facilitated the improvements observed.

## Conclusions

Our results indicate that the six-month Australian TLC Diabetes program led to improvements in diabetes management, with significant benefits to mental health functioning and improved glycaemic control. If these results were maintained long term, such results would be expected to lead to important reductions in diabetes-related complications and mortality [30]. With the increasing accessibility to and feasibility of such telehealth interventions, the TLC program has excellent potential to be ‘scaled up’ and deliverable to large numbers of individuals with diabetes.

## Competing interests

Dr. Friedman has stock ownership and a consulting agreement with Infomedics, the company that owns commercial rights to the TLC technology used in the computerized intervention. He is also a member of its Board of Directors. The other authors declare that they have no competing interests.

## Authors’ contributions

EDW analysed the data and wrote the manuscript. DB collected the data, contributed to study development, discussion and manuscript writing. AF, AR, SA, PS, RF and BO contributed to study development, and discussion, reviewing/editing of the manuscript. All authors read and approved the final manuscript.

## Pre-publication history

The pre-publication history for this paper can be accessed here:

http://www.biomedcentral.com/1471-2458/12/602/prepub
